# Chitosan-Based Particulate Carriers: Structure, Production and Corresponding Controlled Release

**DOI:** 10.3390/pharmaceutics15051455

**Published:** 2023-05-10

**Authors:** Jiaqi Weng, Alain Durand, Stéphane Desobry

**Affiliations:** 1 Université de Lorraine, LIBio, F-54000 Nancy, France; jiaqi.weng@univ-lorraine.fr; 2 Université de Lorraine, CNRS, LCPM, F-54000 Nancy, France; alain.durand@univ-lorraine.fr

**Keywords:** chitosan, controlled release, particles, capsules

## Abstract

The state of the art in the use of chitosan (CS) for preparing particulate carriers for drug delivery applications is reviewed. After evidencing the scientific and commercial potentials of CS, the links between targeted controlled activity, the preparation process and the kinetics of release are detailed, focusing on two types of particulate carriers: matrix particles and capsules. More precisely, the relationship between the size/structure of CS-based particles as multifunctional delivery systems and drug release kinetics (models) is emphasized. The preparation method and conditions greatly influence particle structure and size, which affect release properties. Various techniques available for characterizing particle structural properties and size distribution are reviewed. CS particulate carriers with different structures can achieve various release patterns, including zero-order, multi-pulsed, and pulse-triggered. Mathematical models have an unavoidable role in understanding release mechanisms and their interrelationships. Moreover, models help identify the key structural characteristics, thus saving experimental time. Furthermore, by investigating the close relation between preparation process parameters and particulate structural characteristics as well as their effect on release properties, a novel “on-demand” strategy for the design of drug delivery devices may be developed. This reverse strategy involves designing the production process and the related particles’ structure based on the targeted release pattern.

## 1. Introduction

Chitosan (CS), derived from chitin through deacetylation, is a natural polymer with enormous potential applications in biotechnology and food engineering. Chitin is a long-chain polymer of N-acetyl glucosamine and the second most abundant natural biopolymer after cellulose on the planet [1,2,3,4,5]. The estimated annual biosynthesis of chitin is roughly 1010 tons in the biosphere [6]. It is a sustainable natural resource that is omnipresent but still underexploited commercially. Chitin and cellulose belong to the same class of biopolymers, i.e., polysaccharides. The two other main classes of biopolymers include proteins and nucleic acids.

**Figure 1:**
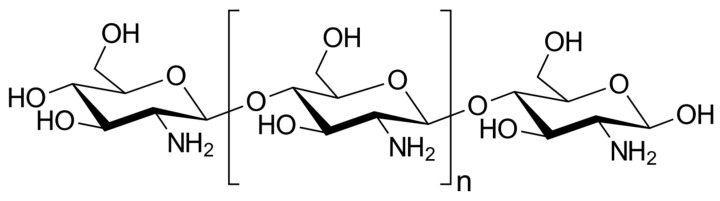
Chemical structure of chitosan from completely deacetylated chitin.

CS is a linear polysaccharide composed of amino groups, with its structure containing D-glucosamine (deacetylated units) and N-acetyl-D-glucosamine (acetylated units) linked randomly through β-(1→4) bonds (Figure 1) [7]. Due to its repeatedly reported beneficial characteristics, such as the absence of toxicity, biocompatibility and biodegradability, CS has found considerable applications in various fields, including environmental engineering, agriculture, aquaculture, agrochemistry, the food industry and the medical/pharmaceutical and cosmetic industries [8,9,10,11].

Chitin can be extracted from algae, fungi, arthropods (crabs, shrimp, crayfish and insects), plankton and mollusks (squids). Nowadays, the main commercial sources of chitin and its derivatives are shells and the exoskeleton of crustaceans, which used to be considered low-value marine wastes. The global annual production of shrimp or lobster shells and crab waste was reported to be between 6 and 8 million tons [12]. For instance, crustacean shells contain roughly 15–40 wt% of chitin [13]. The powder of this grounded marine byproduct can serve as an animal-feed supplement, but with very limited profitability compared with its refined, high-value chemicals (Table 1). The transformation of chitin from marine waste is not complex, but the process requires considerable amounts of water and chemical components such as strong acids and bases. Additionally, the final product quality varies a lot depending on the raw material (species of shellfish as well as types and quantities of impurities).

The industrial production of refined chitin/chitosan and their derivatives remained low by 2016. Indeed, it was reported that less than half of the global demand was satisfied [15,17]. Both demand and production for CS have kept growing in various industries worldwide during the last few years. The global market volume of chitin and its derivatives was valued at nearly USD 7.1 billion by 2021 [18], was estimated at USD 7.9 billion in 2022 and is foreseen to reach a revised size of USD 24.9 billion by 2030 [19].

### 1.1. Emerging Research and Industrial Interest for Chitosan

**Figure 2:**
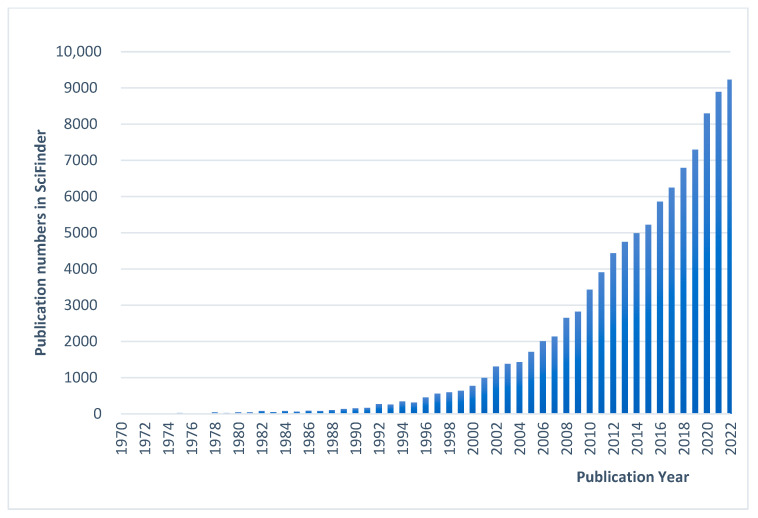
Number of publications containing the keywords “chitin” or “chitosan” registered each year in SciFinder. Books, conferences, editorials, journal articles, preprints and reviews included. (Data from SciFinder, March 2023).

**Figure 3:**
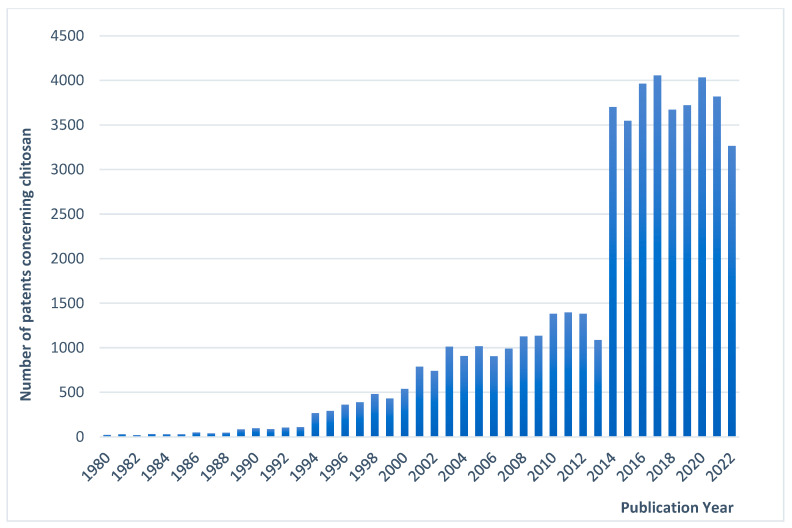
Number of patents relevant to the keywords “chitin” or “chitosan” in English published each year. (Data from WIPO, https://patentscope.wipo.int/search/en/result.jsf/ (accessed on 1 March 2023)).

The first interest in commercializing chitin was held back in the 1930s because of the strong competition with synthesized polymers at that time. Large-scale production of chitin regained attention in the mid-1970s, when regulations aiming to reduce the dumping of shellfish waste were introduced. Regarding research interest, 108,025 references concerning “chitin/chitosan” were found using SciFinder (years between 1970 and 2022). A remarkable increase in the number of publications was evidenced at the beginning of the 1990s, indicating a real emerging research interest in the academic world (Figure 2). On the other hand, according to statistics from WIPO’s database, PATENTSCOPE, the potential commercial applications of CS have grown steadily since the late twentieth century, whereas the number of patent applications concerning CS has exploded in the last three decades. A total of 51,274 patent application records in English were found from all offices (Figure 3). 

### 1.2. A Multiple-Application Biopolymer

Chitosan’s multiple utilities originate from its relatively specific chemical and physical properties. Indeed, it is the unique example of a cationic polyelectrolyte among known natural polysaccharides. Thus, complexes, or coacervates, can be produced through electrostatic interactions between chitosan and other negatively charged compounds. Because of its beneficial biological properties, such as non-toxicity, biodegradability, biocompatibility, mucoadhesive behavior and antimicrobial activities, it is apt to bind with electronegative mucous membranes, and it shows low in vitro toxicity as well as in the case of some in vivo models [20]. CS is also a pH-sensitive material whose dissolution in water is possible only under mildly acidic conditions (pH < 6.5). This may sometimes be considered a limitation. CS derivatives with extended water solubility can be obtained through chemical modification of the chains, such as carboxymethylation, quaternization and hydroxypropylation [21]. There are many examples of functional groups that have been introduced onto the CS chain to form water-soluble derivatives [22], e.g., thiolated CS [23], glycol CS [24,25], quaternized CS [26], carboxymethyl CS [27], isobutyl CS [28] and oligoethylene oxide sulfonate CS [29]. In addition to its well-known applications in various fields (Figure 3), CS has significant potential for managing hyperlipidemia [30]. Recently, there have been reports on the beneficial effects of CS in controlling the COVID-19 pandemic [31,32]. To conclude, CS’s availability (relying on abundant reserves in nature), benign properties and versatile applications rationalize the ongoing research enthusiasm from both academia and industry.

### 1.3. Terminology of Particulate Carriers

Due to chitosan’s versatile properties mentioned above, its application as a delivery system has been reported in numerous papers. At the level of the particulate carrier, CS is commonly used as the principal polymer to build up the carrier’s core material as well as the peripheral material to coat or/and impart novel functionality to the vector.

**Figure 4:**
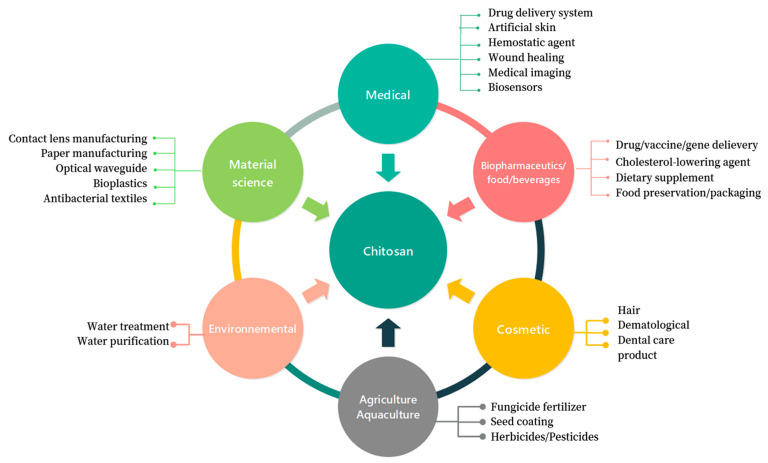
Schematic overview of the versatility of chitosan applications in various fields.

Within the scope of this review, particulate carriers are defined as micro- or nano-sized particulate dispersions of liquid or solid particles. The size range is first related to the route of administration. In addition, the nanometric size range endows this type of object with interesting properties as a delivery system because of the large specific area, which usually facilitates the release of active molecules. Alternatively, their surface is available for further functionalization, providing specific interaction properties potentially leading to targeting. Understanding and characterizing the nature/morphology/size of particles is important for designing and optimizing particle-based systems for specific applications (Figure 4). Additionally, a particle’s shape can have a significant influence on its physical, chemical and biological properties, e.g., surface area, packing properties, flow behavior, mechanical properties, drug release kinetics and efficiency [33,34,35,36]. 

**Figure 5:**
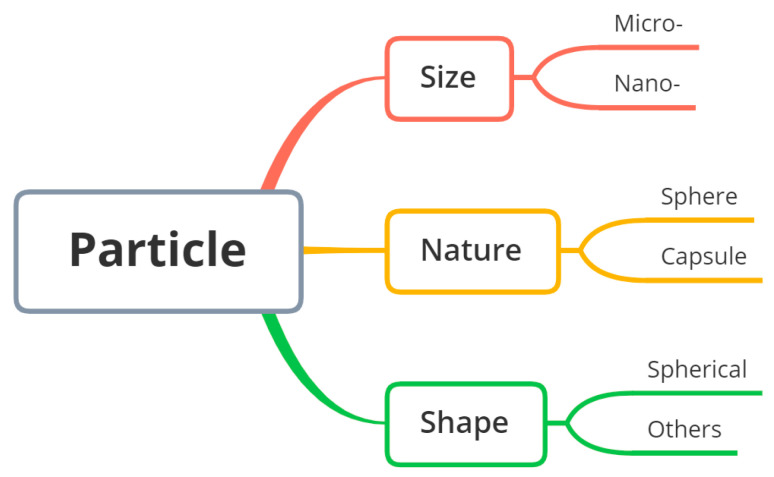
Terminology followed for particulate carriers within the scope of this review.

**Figure 6:**
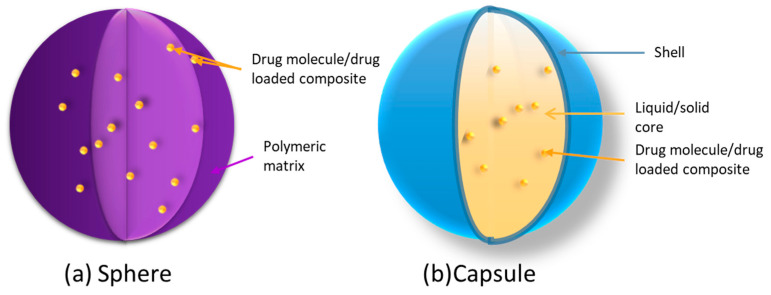
Schematic representation of two types of particulate carriers. (**a**) Polymeric matrix sphere: drug molecules or drug-loaded composites (micro-/nanoparticles) are dispersed both onto carrier surface and into inner sphere matrix. (**b**) Solid/liquid core–shell capsule: drug molecules or drug-loaded composites (micro-/nanoparticles, emulsions, liposomes) are entrapped in the liquid/solid core, which is itself enclosed by a shell-like wall.

A complex nomenclature of particulate carriers exists in the literature [37,38]. Within the scope of this review, prefixes indicate the size of the carrier, such as micro-/nano-. Micro-/nanospheres refer to spherical particles with diameters in the micrometer/nanometer range (Figure 5). According to the International Union of Pure and Applied Chemistry (IUPAC), the lower limit between micro- and nano-sizing is still a matter of debate. This review adapted the terminology of IUPAC (Table 2). A nanoparticle refers to a particle of any shape with at least one dimension between 10^−9^ and 10^−7^ m. The upper limit is chosen as 100 nm because novel properties that distinguish particles from bulk material normally show up at a critical dimension scale lower than 100 nm [39]. Nevertheless, due to certain phenomena (transparency, ultrafiltration, stable dispersion, etc.), the upper limit can be acceptably extended up to 500 nm. Nanoparticles can be divided into two categories: homogeneous nanoparticles, also known as “nanospheres,” and core–shell structured nanoparticles, known as “nanocapsules” (Figure 6) [40,41,42,43].

**Table 2:** Summary of definitions of nano- and microparticles.

Terminology	Concise Definition
Nanoparticle	Particle of any shape with at least one characteristic dimension between 10^−9^ and 10^−7^ m
Nanocapsule	Hollow nanoparticle consisting of a solid shell encircling a core-forming area
Nanosphere	Spherical-shaped nanoparticle without membrane or any distinct outer layer
Microparticle	Particle with at least one dimension between 10^−7^ and 10^−4^ m
Microcapsule	Hollow microparticle composed of a solid shell surrounding a core-forming space
Microsphere	Microparticle of spherical shape without membrane or any distinct outer layer

A nano-/microsphere is composed of a matrix where substances can be permanently or temporarily embedded, dissolved or covalently bound. Nano-/microcapsules are submicroscopic colloidal drug carrier systems composed of an oily/aqueous core surrounded by a thin membrane, which is usually, but not necessarily, made of polymer [39].

From a toxicological and pharmaceutical perspective, vesicular capsules possess an advantage over matrix spheres because of their lower polymer content and high loading capacity for both hydrophilic and lipophilic active molecules [41].

## 2. Preparation Methods for Chitosan-Based Network and Particulate Structure

Over the last few years, many attempts have been made to develop synthetic methods for CS-based particles, such as ionic gelation [44,45,46], polyelectrolyte complexation [47,48,49], emulsion solvent diffusion [50,51], emulsion crosslinking [52,53], spray drying [54,55], supercritical fluid drying [56], electrospraying [57], emulsion droplet coalescence [58], reverse micellar/emulsion methods [59,60] and sieving methods [61]. 

**Figure 7:**
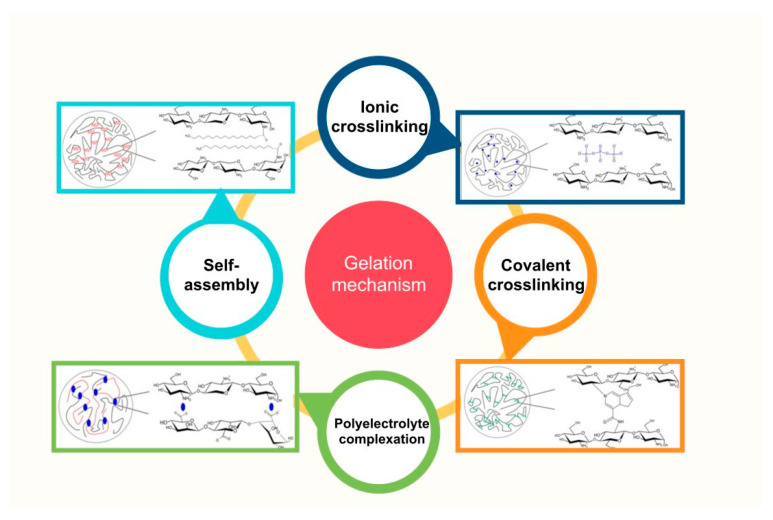
Chitosan crosslinking gelation mechanisms (Adapted from [62] Copyright 2016, Bellich et al.)).

Due to its mild reaction conditions and simple process, the crosslinking gelation method has been extensively studied. The gelation method usually generates the polymer matrix structure, the coating/shell or the core region of core–shell capsules. There are several CS crosslinking gelation mechanisms that offer numerous possibilities for preparing fine-tuned particulate carriers (Figure 7) [62]. Apart from the required experimental conditions, the various gelation methods differ by the nature of the inter-chain crosslinks (ionic, hydrophobic association, covalent, etc.) and, consequently, their stability, reversibility and timescale, among others.

Schematically, spray drying and supercritical processes can produce monolithic matrix (sphere)-structured particles, while capsule structures can be produced via emulsification (emulsion–alkali coacervation/precipitation, emulsion–emulsion coacervation method), electrospraying and microfluidic processes. Table 3 summarizes frequently used methods for the preparation of CS-involved particulate carriers, their main advantages and shortcomings, the size range of the obtained particles and, when available, a rough estimation of the particle concentration at the process outlet.

The structure of a particulate carrier is strongly related to its preparation method. Therefore, selecting the most suitable preparation process is strongly related to the required structural characteristics and, thus, the targeted application. Process parameters, including pH, temperature, concentration of reagents, mass ratio of polymer and crosslinker/surfactants, nature of colloidal stabilizers and agitation speed, have a strong effect on the carrier’s structural properties, such as particle average size, particle size distribution, particle shape, porosity, swelling capacity, degradation rate and diffusivity of the drug through the carrier material [63]. Numerous research studies have explored examples of this relationship using the Design of Experiments (DoE) method [64,65,66].

**Figure 8:**
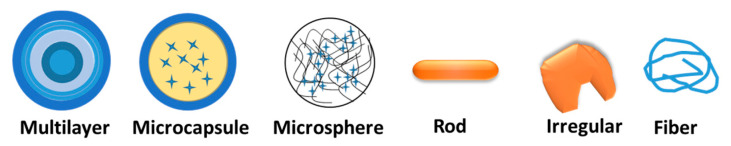
Schematic illustration of chitosan-based particles with various structures.

For each method reported in the literature, some examples of developed particulate systems were selected and briefly described, along with information about particle size range, morphology of particles (Figure 8), geometrical characteristics of particles and particle concentration. 

The parameter “particle concentration” was defined as the concentration of newly-fabricated particles in their dispersing medium after formation but before any extra operations such as separation or dilution. This concentration was calculated or estimated whenever allowed by the provided experimental data. This parameter may be important regarding the scale-up or transfer to industrial processes of lab-scale preparation procedures. Indeed, when very dilute suspensions of particles are produced, further steps may be necessary to increase particle concentration before application. 

**Table 3:** Preparation methods of chitosan relevant particles. Advantages and shortages of each method and geometric dimension of obtained particles. The cartoon symbols present structural characteristics of particles.

Method	Description	Comments		Particle Dimension	Geometry of Particle	Particle Concentration (*w*/*v*)	Key Parameters	References
		Merit (s)	Demerit (s)					
Droplet extrusion method	Droplets of drug-loaded polymeric solution are formed by extrusion through a nozzle into a bath of an aqueous solution of polyvalent cations.	The process is convenient, cost-effective and devoid of high temperatures and use of solvent. Also usable for living-cell encapsulation.	Limited size control/size reduction. Difficulties in large-scale production. Teardrop-shaped particles.	90 µm–7 mm	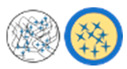	-	Polymer concentration; viscosity of polymer solution; flow rate; geometry of extrusion device; type and concentration of non-solvent bath.	Microcapsule [67]; microsphere [68].
Crosslinking gelation	Electrostatic interaction between polyelectrolytes and polyvalent ions is often used as the driving force to form micro-/nanoparticles. The positively charged natural polymer CS has been broadly investigated to form composites with negative electrolytes by ionic crosslinking (ionotropic gelation). Alternatively, covalent crosslinking has been used.	Mild processing conditions. Simple equipment. Ionotropic gelation: low toxicity, limited risk of altering the encapsulated drug.	Poor stability in non-acidic conditions. Difficulty in encapsulating high-molecular-weight drugs. Toxicity of certain covalent crosslinkers (aldehydes, for instance).	10 nm–3 mm	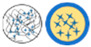	0.02–1.2%	Polymer concentration; crosslinking agent concentration; mixing rate and time; temperature; pH.	Microsphere [45,46,65,69,70,71,72,73,74,75,76,77,78,79,80,81,82]; microcapsule [83,84].
Polyelectrolyte complexation (PEC)	Complexation of CS with synthetic anionic polyelectrolytes or natural anionic biopolymers via electrostatic interaction.	Able to encapsulate macromolecules such as polypeptides and polynucleotides, as well as hydrophobic drugs.	Toxicity of certain covalent crosslinkers (aldehydes, for instance).	50–450 nm	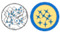	0.1–2%	Polyelectrolyte concentration; pH; mixing rate and time; temperature; solvent type.	Microsphere [76,84,85]; microcapsule [86].
Complex coacervation/precipitation	CS acetic solution was mixed up with a DNA/protein dissolved salty (sodium sulfate) solution to form micro-/nanospheres.	Narrow particle size distribution; high encapsulation efficiency; relatively low cost of processing.	Safety issue of toxic crosslinkers; poor product formation due to poor solubility of active agent (e.g., plant protein).	50–1600 nm	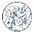	0.25–0.7%	Nature and concentration of polyelectrolytes; pH; temperature; solvent and co-solvent.	Microsphere [76,87,88].
Emulsion–coacervation (Emulsion–alkali precipitation) method	Drug/oil mixture is dispersed in CS acidic solution under stirring, followed by ultra-sonication/homogenization to obtain homogeneous emulsion. Microcapsules were obtained by dropping alkaline solution into aforesaid emulsion.	Devoid of crosslinker.	Suitable for lipophilic drug encapsulation.	10–12 µm	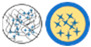	0.05–0.19%	Type and concentration of the polymer, surfactant and alkaline solution; emulsion stirring rate; aging time.	Microcapsule [52,89,90]; double-walled microspheres [78].
Emulsion crosslinking	CS aqueous solution is dispersed into oily phase in the presence of suitable surfactants as emulsion stabilizers. Thermal crosslinking produces microspheres.	Mild processing conditions.	Complete removal of the unreacted crosslinking agent may be difficult due to possible toxicity.	30–700 µm	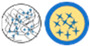	0.05–1.66%	Polymer concentration; crosslinking agent concentration; mixing speed and time; temperature; pH.	Microspheres [53,91,92,93,94]; microsphere-loaded core–shell carrier [50,95].
Emulsion solvent diffusion method	An o/w emulsion is prepared by mixing organic solvent into a solution of CS with stabilizer under mechanical stirring, followed by high-pressure homogenization/ultra-sonication. Add a large amount of water to the emulsion to form particles.	High encapsulation efficiency of hydrophobic drugs.	High shear force involved in the process; use of organic solvent.	0.1–45 µm	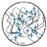	2%	Solvent selection; emulsification conditions: stirring rate, emulsifying time and temperature.	Micro-/nanosphere [96,97].
Spray-drying method	CS is first dissolved in an aqueous medium, and then the drug is dissolved or dispersed in the previous solution. Crosslinker is added to the polymeric solution. Particles are produced by atomization and subsequent solvent evaporation.	Low impact on the solubility of drug and polymer; simple, reproducible and easy to scale up.	Degradation due to high temperatures or/and high shear rates during atomization.	0.2–60 µm	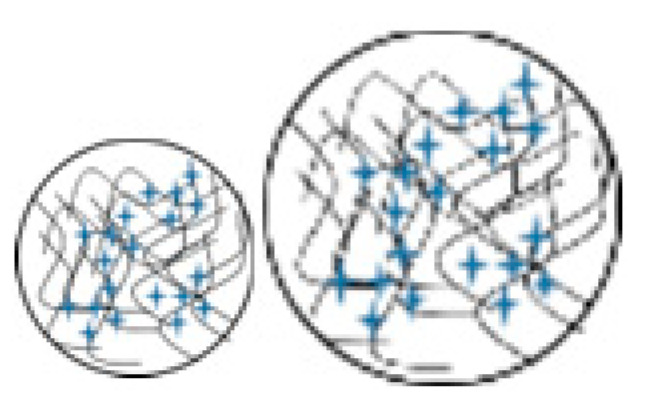	-	Feed composition and concentration; operation temperature; flow rate and pressure of the atomizing air; spray rate; drying time; type and concentration of the surfactant.	Micro-/nanosphere [54,98,99,100,101,102].
Supercritical technique	Microspheres are fabricated by spraying a drug-loaded HCL/DMSO solution into supercritical carbon dioxide.	Small-sized particles (<3 µm); fast; cost-effective.	Rather broad particle size distribution.	0.4–10 µm	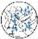	-	Temperature and pressure of the supercritical fluid; solvent type and concentration; flow rate; nozzle geometry; antisolvent addition.	Nanosphere [56]; microsphere [103].
Electrospraying	CS is dispersed/dissolved into a mixture of solvent and blend with drug solution/suspension. The conductive liquids are atomized under high voltage to form drug-encapsulated particles. The flow rate, voltage and distance between needle tip and collector are crucial process parameters.	Low production cost; narrow particle size distribution; easy-to-control surface properties and rapid preparation; high drug-loading efficiency; gentle conditions without use of harsh solvents.	Further investigation needed for upscaling; potential toxicity due to certain solvents.	0.1–1.3 µm	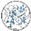	-	Flow rate; solvent evaporation rate; collector distance; electrical conductivity; nature of polymer, solvent and molecules being used in the process.	Nanosphere [57,104,105].
Reverse microemulsion/micellar method	Organic solvent (containing surfactant) is mixed with acidic CS solution to form reverse micelles. Then, drug conjugate and CS attach to the micelles via glutaraldehyde (crosslinker) to form nanoparticles. Residual solvent and surfactant and excess crosslinking agent need to be removed.	Ultrafine particle size (<100 nm); narrow particle size distribution.	Application of organic solvent; time-consuming preparation process; complex washing step.	60–130 nm	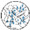	0.01–0.1%	Choice of surfactant and co-surfactant; type and concentration of oil phase; water-to-oil ratio; temperature and stirring speed; addition of crosslinking agents.	Nanosphere [59,60,106].
Sieving method	A drug-loaded CS jelly mass is crosslinked and then manually passed through a sieve to obtain non-sticky particles.	Simple and commercially viable; easy scale-up; devoid of tedious processes; high drug loading.	Irregular particle shape.	500–600 µm	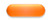 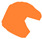	-	Mesh size of the sieve; amplitude and frequency of vibration; duration of the sieving process; properties of the material.	Rod- or irregular-shaped microparticles [61].
Solvent displacement/interfacial deposition method	Sub-microcapsule nanoemulsion coated with CS shell.	Suitable to encapsulate lipophilic drugs; rapid and easy operation; narrow size distribution; absence of shearing stress.	Use of organic solvents.	130–500 nm	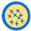	0.1–0.33%	Polymer concentration; selection of solvent; selection of non-solvent; mixing rate; temperature; surfactant concentration; pH; addition rate.	Nanocapsule [107,108,109].
Microfluidic technique	Dispersed phase and continuous phase are syringe-pumped onto microchannel of the microfluidic chip to obtain droplets, which are subsequently hardened by precipitation or crosslinking.	Well-controlled size; able to entrap hydrophilic and/or lipophilic molecules; controllable particles.	Low production rate; difficulties in mass production (scale-up) except parallelization; high cost.	0.2–600 µm	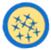 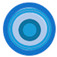 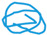	-	Flow rate; viscosity; temperature; device geometry; electrical and magnetic fields.	Microcapsule [47,110,111]; multilayer particle [81,112,113]; microfiber [114].

## 3. Characteristics of Chitosan-Involved Particulate Carrier

The morphology of sub-microparticles is a fundamental characteristic that significantly affects their properties. Several characteristics of nano-/microparticles are essential to know, such as average size and size distribution, shape, surface properties (area, charge, functionalization), porosity, etc. These properties are desirable for assessing safety, ensuring consistent product quality control and ensuring regulatory compliance. 

The size distribution of spherical partials is a subject that has been well illustrated and developed. Briefly, it is generally required to combine light-scattering techniques (DLS, LS) with microscopic characterization using TEM, SEM and AFM [115,116,117,118]. However, for non-spherical particles, the diffusion coefficient also depends on the shape of the particles [119,120]. A combination of several techniques is recommended to obtain precise information on particle size and shape. For example, particles with irregular shapes obtained from the sieving method have been characterized by the laser light diffusion method and SEM micrographs. LS was used to determine the size and distribution of spherical particles equal in volume to the samples [61]. As for rod-shaped and cylindrical particles, the aspect ratio was introduced to describe the elongation of the particle shape [119]. The characterization of fiber-shaped particles requires more complex techniques as they have an elongated shape and cannot be adequately described by a single dimension. Size characterization of fiber-shaped particles can be conducted based on various dimensions such as diameter, length, aspect ratio and specific surface area [120,121,122,123]. Finally, it must be reminded that average values of particle size may differ from one technique to another simply because “averages” are not calculated in the same way (number, surface, volume, intensity, etc.).

Additionally, Small-angle X-ray scattering (SAXS) is a technique that can be used to determine the size, size distribution, shape and organization of hierarchal structures [124]. SAXS is based on the interaction of X-rays with the electrons in the material, producing scattering patterns that can interpret particle shapes such as spheres, rods, discs, hollow spheres and dumbbells [125]. However, SAXS is limited to analyzing samples in the range of 1–100 nanometers, and interpreting SAXS data, especially when the sample is complex or contains multiple components, could be challenging. 

As to capsule and multilayer particles, besides the size of the particle (the diameter of the outermost shell), the thickness of the layer(s) (including shell thickness) is also an interesting characteristic to know since the properties of layers made of different materials can be pretty diverse. However, this characteristic has not been abundantly discussed in the literature.

**Figure 9:**
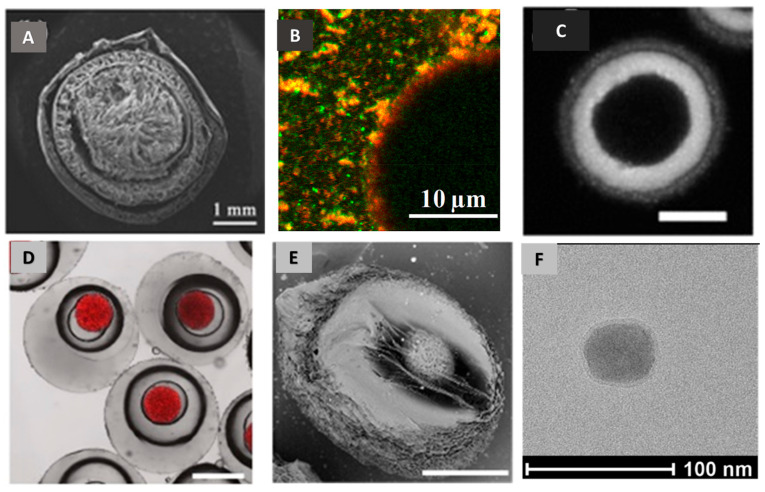
Internal structure and layer thickness of particles can be characterized by SEM, CLSM and TEM images. (**A**) Cross-sectional morphologies (SEM) of three-layer CS hydrogel capsules (adapted with permission from Ref. [81]. Copyright 2019, Elsevier); (**B**) CLSM picture of fresh oil-in-water CS emulsion (adapted with permission from Ref. [131]. Copyright 2020, Elsevier); (**C**) CLSM fluorescence image of CS-coated microcapsule, scale bar = 5 μm (adapted with permission from Ref. [132]. Copyright 2012, Wiley); (**D**) CLSM image of O1/W2/O3/W4/O5 microcarriers; (**E**) SEM image of dual-responsive microcarriers without O1 and O3 cores. Scale bars are 200 μm, adapted with permission from Ref. [112]. Copyright 2019, Elsevier; (**F**) TEM image of CS nanoparticle with particle size of around 45 nm and silica shell thickness of 5 nm (adapted from Ref. [133]. Copyright 2019, Arzumanyan G, et al.).

Microscopy techniques (SEM, TEM, CLSM) play a significant role not only by visualizing the surface morphology, shape and size of sub-microparticles but also their internal structure (cross-section, porosity, crystallinity) [113,126,127,128,129]. Additionally, the structural evolution of particles during the release process can be monitored by consecutive micrographs, which can reveal and confirm the release mechanism over time [130]. SEM and TEM can provide high-resolution images of the particle structure and morphology, allowing for direct visualization of the particles at the nanoscale. Confocal microscopy uses a focused laser beam to scan a sample and create a series of optical sections at different depths. Confocal slices can provide detailed information about the internal structure and organization of the sample at a particular depth or plane (Figure 9). By using fluorescent labeling, the oil phase inside the core of microcapsules was localized and quantified [130]. The fluorescence signals of polymers allow the visualization of their distribution within the polymeric shell. Furthermore, the oil phase is distinguished unambiguously from air bubbles by comparing optical and fluorescent images. With the help of computational image analysis, the layer thickness and the volumes of different phases can be estimated.

## 4. Particulate Structure and Controlled Release Kinetics

Drug release refers to the process by which entrapped drugs dissolve and diffuse into the outer medium by diffusing within bulk core material and/or shell material or passing through pores or fractures within the particles. Drug release kinetics depend greatly on the particulate building materials, drug properties and structural properties of composites, including shape, particle size, surface roughness, porosity, shell thickness, etc. Additionally, along the release process, carrier structure may evolve under the effect of stimuli in the release environment. The assumed principal drug release mechanisms include dissolution, erosion, swelling and diffusion [20,134]. Release mechanisms and the corresponding release profiles dominated by each were summarized in Ref. [62]. To simplify the analysis of the experimental release results, it is generally crucial to identify the limiting phenomena. 

**Figure 10:**
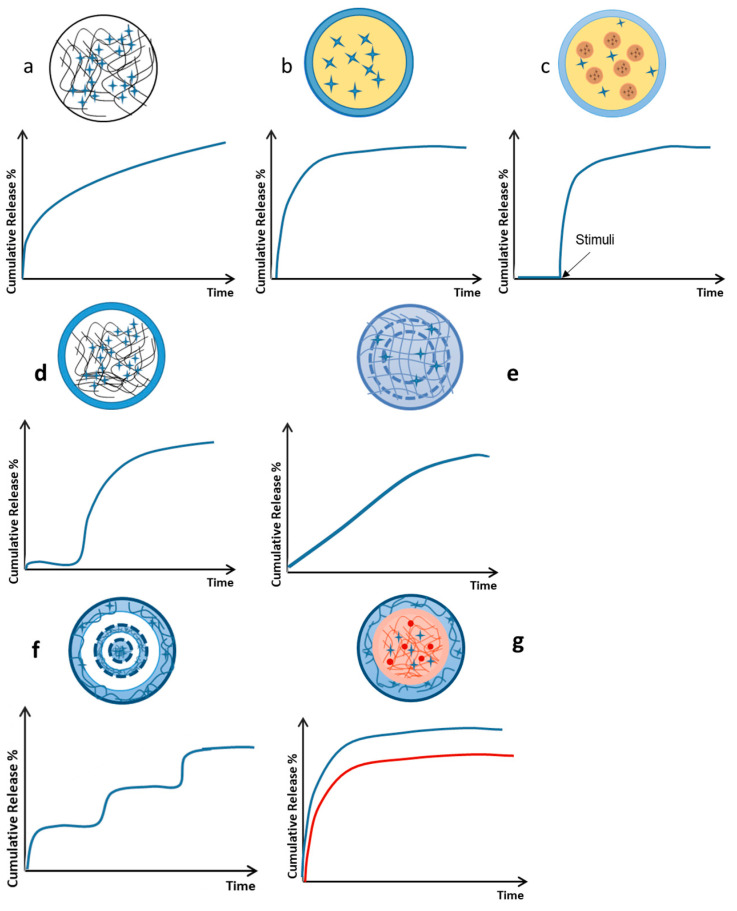
Schematic illustration of typical chitosan sub-microparticulate carriers and corresponding release profiles found in the literature. The shuriken-shaped blue marks and red dots stand for active ingredients loaded. (**a**) Monolithic sphere; (**b**) capsule with liquid core; (**c**) nanoparticle-loaded capsule with CS shell; (**d**) core–shell sphere with CS matrix core; (**e**,**f**) multilayered CS hydrogel capsule; (**g**) core–shell sphere loading two drugs.

As the drug release process results from interactions between entrapped molecules, encapsulating particles and the releasing environment, the particulate structure and its evolution over a definite release time have a great effect on release kinetics. Elucidating the corresponding relations between composite structures and potential release mechanisms enables researchers to predict the release trend of certain loaded active ingredients from a specific structured vehicle. Figure 10 summarizes graphically several typical CS-involved particulate carriers with relevant release profiles found in the literature. In Figure 10a, which illustrates CS polymeric matrix particles, drug molecules are distributed on the particle surface as well as inside the matrix. The diffusion of molecules located superficially may lead to a burst release, i.e., the fast release of a significant number of loaded molecules before the further and slower release of the remaining substance. Medium permeation inward the carrier causes erosion, swelling and diffusion, which are mainly responsible for the following sustained release, which may last from hours to days [83,111]. Figure 10b represents a core–shell capsule case. The core region could be solid or liquid (oil phase), which encapsulates dissolved active ingredients or dispersed systems such as emulsion droplets, nano-/microparticles or liposomes [82,112,135]. The outer wall-like shell prevents leakage and the degradation of the inner contents from harsh conditions inside the internal environment, such as pH, enzymes, etc. Structural incompleteness due to fracture or breakage of the shell leads to the liberation of inside molecules. Figure 10c is a core–shell-structured microcapsule encapsulating drug-loaded nanoparticles in an oily core enclosed by a CS shell. Both drug molecules and drug-loaded poly-(lactic-co-glycolic acid) (PLGA) nanoparticles were enclosed in a stimulus-responsive microcapsule [110]. The CS shell prevented the leakage of the entrapped cargo in a neutral medium and broke down in an acidic site, thus providing sustained drug delivery through the diffusion of free drug and nanoparticle degradation. Additionally, enzyme intestinal delivery was reported to be localized by alginate nanoparticles incorporated into CS-shelled microcapsules [113]. Figure 10d is a CS-based microsphere with an alginate coating. The coating can protect the loaded substance from degradation and hydrolysis in acidic conditions for hours and can modulate the release rate by suppressing burst release [103]. In addition, in some other core–shell cases, the external shell is able to respond to certain stimuli, e.g., pH and ionic strength [82], and achieve targeting effects through the addition of biological ligands [84]. Figure 10e,f are both multilayered CS hydrogel capsules. The drug is loaded homogenously in each layer of the carrier in Figure 10e, while the one in Figure 10f contains sequentially alternating drug-loaded and void layers. It is possible to customize the number of layers and tailor their thickness [136]. The former achieves approximately a zero-order release, while the latter is supposed to attain a pulsatile drug release [81]. Apart from CS-based carriers alone, the encapsulated ingredient is also a dimension that can enrich the utility of these functional carriers. For example, Figure 10g represents a core–shell nanosphere system that was developed for co-delivering drugs (oleanolic acid and doxorubicin) as a strategy to treat multi-drug-resistant breast cancer. This novel dual-drug-loaded DDS was proven effective as a breast-tumor targeting strategy in in vitro and ex vivo evaluations [137]. Below are some typical CS-relevant carriers with their release profiles found in the literature. Particularly worth mentioning is the fact that certain release profiles may be attained by diverse carriers. Inversely, a carrier may possess different release profiles under different dissolution conditions (pH, ionic strength, light, temperature, magnetic field, etc.). 

To sum up, further investigations into the correlation between carrier structure and the associated release profile are worth the effort. By achieving this goal, in turn, it would be possible to design on-demand drug delivery systems that can regulate, in an expected way, more precisely the release rate of certain drugs at a specific time interval and location.

## 5. Release Kinetics, Mechanisms and Modeling

**Figure 11:**
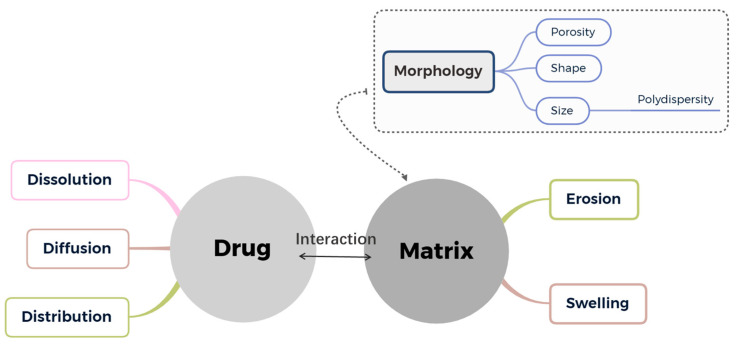
Factors influencing drug release kinetics.

The use of kinetic models can aid in describing the release rate of drugs, leading to increased efficiency, accuracy and safety of the dose. This, in turn, can help optimize the design of drug delivery devices [138]. With an appropriate understanding of the limiting phenomena that govern drug release from a given system, it is possible to describe drug release behavior by applying proper mathematical models. As known, various factors greatly influence this complex process, such as matrix geometry, matrix swelling equilibrium and kinetics, matrix erosion, drug dissolution and partitioning, drug diffusion, drug–matrix interaction, initial drug distribution, etc. (Figure 11). Based on specific assumptions and hypotheses, certain mathematical models enable the simulation of release kinetics under certain conditions. Some of these mathematical models have been commonly used to identify dominant release mechanisms on the basis of the comparison between experimental and theoretical time variations in cumulated released amounts [138]. 

Trying to develop a general and unifying model would consequently result in an increase in the complexity of the model’s expression and make it difficult to obtain analytical and/or numerical solutions. Although achieving a highly general model may be challenging, researchers have explored the potential of using empirical/semi-empirical models to describe the release kinetic characteristics, prioritizing various aspects that make up the release phenomenon. Mathematical models used to fit the drug release profile from CS-based particles include the Higuchi square root, Korsmeyer–Peppas’, Hixon–Crowell’s, Baker–Lonsdale’s, Peppas–Sahlin’s, Kopcha’s, Hopfenberg’s and Gallagher–Corrigan (GC) models. According to the systems, zero-order or first-order kinetics may be observed.

For zero-order kinetics, the release of an active agent is only a function of time, and the process takes place at a constant rate independent of active agent concentration:

(1) $$M_{t}=kt$$

where *M_t_* is the amount of drug released at time *t* and *k* is the zero-order constant.

The first-order release kinetics model assumes that the rate of drug release is proportional to the amount of drug remaining in the dosage form [139,140]:

$$1-\frac{M_{t}}{M_{0}}=e^{-k^{\prime }t}$$

where *k*′ is the first-order rate constant and *M*_0_ is the initial amount of drug in the dosage form.

The Higuchi model explains the release of a drug as a diffusion process, which is governed by Fick’s law and has a time-dependent square root relationship [141]:

(2) $$\frac{M_{t}}{M_{\infty }}=k_{H}\sqrt{t}$$

where *M_∞_* is the absolute amount of drug released over infinite time and *k_H_* is a release rate constant.

The Korsmeyer–Peppas model is a semi-empirical model that establishes the exponential relationship between the amount released and the time and provides indications about the mechanism of drug release [142,143,144]:

(3) $$\frac{M_{t}}{M_{\infty }}=Kt^{n}$$

where *K* is a rate constant and *n* is the exponent that incorporates the effects of the release mechanism and the geometrical characteristics of the system.

The Hixson–Crowell model was initially derived from a work dealing with agitation [145]:

(4) $$\sqrt[{3}]{M_{0}}=\sqrt[{3}]{M_{0}-M_{t}}+K_{HC}t$$

where *K_HC_* is the constant of incorporation, which relates surface and volume.

The Peppas–Sahlin model assumes that drug release occurs through diffusion with a simultaneous influence of polymer relaxation and that it is possible to include the two contributions by summation (diffusional and relaxational terms) [146]:

(5) $$\frac{M_{t}}{M_{\infty }}=k_{d}t^{m}+k_{r}t^{2m}$$

where *k_d_* and *k_r_* are the kinetic constants of diffusion and relaxation, respectively, and *m* is the diffusion exponent.

Kopcha’s model [147], also relying on the assumption of summation, includes simultaneous diffusion and erosion contributions within the release kinetics:

(6) $$M_{t}=Bt+A\sqrt{t}$$

where *A* and *B* are the diffusion constant and erosion constant, respectively.

Based on the Higuchi model, the Baker–Lonsdale model was developed for controlled drug release from spherical matrices [148]:

(7) $$\frac{3}{2}\left[1-{\left(1-\frac{M_{t}}{M_{\infty }}\right)}^{\frac{2}{3}}\right]-\frac{M_{t}}{M_{\infty }}=k^{\prime \prime }t$$

where *k*″ is the release constant, which corresponds to the slope of the experimental curve [149,150]. This equation can be utilized for the linearization of release data for many microparticle formulations [143].

Hopfenberg derived a model to explain the release of drugs from an erodible system in the case of a spherical particle [151]:

(8) $$\frac{M_{t}}{M_{\infty }}=1-{\left[1-\frac{k_{0}t}{C_{0}a_{0}}\right]}^{3}$$

where $k_{0}$ is the erosion rate constant, $C_{0}\;$ is the initial concentration of drug in the matrix and $a_{0}\;$ is the initial radius. 

The Gallagher–Corrigan (GC) model was applied to evaluate drug release from biodegradable polymeric drug delivery systems by combining diffusion and polymer relaxation/degradation contributions:

(9) $$\frac{M_{t}}{M_{\infty }}=A_{1}\left(1-e^{-k_{1}t}\right)+A_{2}\;\frac{e^{k_{2}\left(t-t_{m}\right)}}{1+e^{k_{2}\left(t-t_{m}\right)}}$$

where $A_{1}$ and $A_{2}$ are constants related to the contributions of the diffusion and relaxation mechanisms to drug release, $k_{1}$ is the release constant in the first stage, $t_{m}$ is the maximum release time and $k_{2}$ is the release constant during the stage of polymer degradation.

The GC model is particularly useful for predicting drug release profiles under different conditions [152]. Additionally, it can be adapted to describe dual-phased drug release [152,153,154]. The experimental data on the release of curcumin from MnFe_2_O_4_ magnetic nanoparticles with multilayered CS–alginate (ALG) shells were a good fit to the GC model. The CS-ALG coating was reported to be useful in inhibiting burst release, and the increase in the number of layers could delay the dissolution rate, thus achieving sustained release [154].

The distinguishing aspect of the last three models from others is their incorporation of two discrete phenomena that occur during drug release: diffusion and relaxation in the Peppas–Sahlin and Gallagher–Corrigan models and diffusion and erosion in Kopcha’s model.

Frequently used models are adopted to describe the release characteristics and mechanisms of drugs from CS-based systems, prioritizing different factors (Table 4).

It is important to note that good-fitting experimental data are necessary but may not be sufficient to identify the best model among different models devoted to correctly describing complex release kinetics. Mathematical hypotheses are supposed to take experimental observation of phenomena into consideration. However, in practice, it may not always be feasible. For instance, swelling and erosion of the CS matrix also contributed to the release of vitamins, but the best model chosen was the Peppas–Sahlin model, which privileges more diffusion and relaxation [158]. By synthetically analyzing characteristic constants of diverse models, researchers can better understand the underlying mechanisms of drug release and predict the release kinetics more accurately [1].

Furthermore, considering the overall size distribution of particles rather than just the unitary average diameter may be crucial in developing more accurate models, as heterogeneity in samples is a common occurrence in practical applications [162,163].

It is noteworthy that the systematic implementation of mathematical modeling in the development of active ingredient delivery systems can enhance R&D efficiency, save time and reduce expenses. Additionally, the advanced utilization of this approach can enable the advancement of precision medicine. Instead of an undifferentiated regular dosage regimen, personalized medicine can afford a better therapeutic effect and lower toxicity by considering patients’ individual characteristics.

## 6. Conclusive Remarks and Prospective Research

Tremendous interest in CS from both academic and diverse industrial fields has emerged over the past few decades. Innovative carriers have been developed with unique properties such as sustained release, responsiveness to environmental factors and multiphase release.

An improved understanding of the close relationship between the preparation process and particle structure would enable the prediction of formulation and preparation strategies for drug delivery systems to achieve the desired release kinetics [152,164]. Additionally, modeling capacity is a powerful tool to increase the efficiency of developing new systems, which is crucial for the industry to turn the idea of on-demand drug delivery systems (DDS) into a reality.

Efforts should be focused on investigating in a more specific manner how the preparation process affects the structure of the vehicle and subsequent release behaviors from the polymeric network. By gaining more insight into these aspects, it is possible to design DDSs that release drugs in a controlled manner, such as sustained release, pulsatile release or targeted release, depending on the specific therapeutic needs.

## Figures and Tables

**Table 1:** Global annual production and price of chitin, chitosan and dried shrimp shells.

Product	Industrial Production (10^6^ Tons Per Year)	Price (USD Per Ton)
Dried shrimp shells	6–8	100–120 [14,15]
Chitin	0.02–0.04 ^i^	6000–40,000
Chitosan	<0.2 ^ii^	15,000–160,000 ^iii^

^i^ Annual production of chitin is probably under 10,000 tons, whereas more recent figures are not available [14]. ^ii^ Global industrial production of chitosan is estimated to reach 173.9 thousand tons by 2027 [9]. ^iii^ Data from Alibaba (March 2023) [16].

**Table 4:** Mathematical models to reveal the release mechanisms of reported chitosan-based particulate systems.

Mechanism (s)	Description of Systems	Model	Equation(s)	References
Diffusion	Crosslinked CS-dextran sulfate nanoparticle	Higuchi	(2)	[85]
	Crosslinked CS microspheres	Korsmeyer–Peppas/Higuchi	(3)(2)	[94]
	Spray-Dried CS Microspheres	Higuchi/Korsmeyer	(2)(3)	[155]
	CS hydrogel	Zero-order kinetic	(1)	[156]
	Fatty acid-grafted CS hydrogel	Higuchi	(2)	
	CS-LLA ^i^	Hixson	(4)	
	CS–alginate nanoparticles	Korsmeyer–Peppas	(3)	[157]
Diffusion and relaxation	CS–genipin matrices	Peppas–Sahlin	(5)	[158]
	Multilayer CS–alginate-coated nanocarrier	Gallagher–Corrigan	(9)	[154]
	Alginate–carboxymethylcellulose microparticles with CS shells	Gallagher–Corrigan	(9)	[152]
Diffusion and swelling	CS–alginate	Hopfenberg	(8)	[159]
Diffusion, erosion	DOX-loaded PLGA-QCS ^ii^ core–shell polymersomes	Korsmeyer-Peppas	(3)	[160]
		Kopcha	(6)	
	PLGA/CS microcapsules	Baker–Lonsdale	(7)	[161]

^i^ LLA: linolenic acid; ^ii^ QCS: Chitosan Quaternary Ammonium Salt.

## Data Availability

Not applicable.
